# Chitosan-Enriched Solution Blow Spun Poly(Ethylene Oxide) Nanofibers with Poly(Dimethylsiloxane) Hydrophobic Outer Layer for Skin Healing and Regeneration

**DOI:** 10.3390/ijms23095135

**Published:** 2022-05-05

**Authors:** Emilia Szymańska, Michał Wojasiński, Robert Czarnomysy, Renata Dębowska, Iwona Łopianiak, Kamil Adasiewicz, Tomasz Ciach, Katarzyna Winnicka

**Affiliations:** 1 Department of Pharmaceutical Technology, Medical University of Bialystok, Mickiewicza 2c, 15-222 Białystok, Poland; kwin@umb.edu.pl; 2 Faculty of Chemical and Process Engineering, Warsaw University of Technology, Waryńskiego 1, 00-645 Warsaw, Poland; michal.wojasinski@pw.edu.pl (M.W.); iwona.lopianiak.dokt@pw.edu.pl (I.Ł.); tomasz.ciach@pw.edu.pl (T.C.); 3 Department of Synthesis and Technology of Drugs, Medical University of Bialystok, Kilińskiego 1, 15-089 Bialystok, Poland; robert.czarnomysy@umb.edu.pl; 4 Dr Irena Eris Centre for Science and Research, Armii Krajowej 12, 05-500 Piaseczno, Poland; renata.debowska@drirenaeris.com; 5 Doctoral School No. 1, Warsaw University of Technology, Plac Politechniki 1, 00-661 Warsaw, Poland; 6 Student Scientific Group, Department of Pharmaceutical Technology, Medical University of Bialystok, Mickiewicza 2c, 15-222 Białystok, Poland; adasiewicz.k@outlook.com; 7 Centre for Advanced Materials and Technologies (CEZAMAT), Warsaw University of Technology, Poleczki 19, 02-822 Warsaw, Poland

**Keywords:** chitosan, nanofibers, solution blow spinning, poly(ethylene oxide), wound healing, excised human skin, fibroblast migration

## Abstract

Chitosan (CS)/poly(ethylene oxide) (PEO)-based nanofiber mats have attracted particular attention as advanced materials for medical and pharmaceutical applications. In the scope of present studies, solution blow spinning was applied to produce nanofibers from PEO and CS and physicochemical and biopharmaceutical studies were carried out to investigate their potential as wound nanomaterial for skin healing and regeneration. Additional coating with hydrophobic poly(dimethylsiloxane) was applied to favor removal of nanofibers from the wound surface. Unmodified nanofibers displayed highly porous structure with the presence of uniform, randomly aligned nanofibers, in contrast to coated materials in which almost all the free spaces were filled in with poly(dimethylsiloxane). Infrared spectroscopy indicated that solution blow technique did not influence the molecular nature of native polymers. Obtained nanofibers exhibited sufficient wound exudate absorbency, which appears beneficial to moisturize the wound bed during the healing process. Formulations displayed greater tensile strength as compared to commercial hydrofiber-like dressing materials comprised of carboxymethylcellulose sodium or calcium alginate, which points toward their protective function against mechanical stress. Coating with hydrophobic poly(dimethylsiloxane) (applied to favor nanofiber removal from the wound surface) impacted porosity and decreased both mechanical properties and adherence to excised human skin, though the obtained values were comparable to those attained for commercial hydrofiber-like materials. In vitro cytotoxicity and irritancy studies showed biocompatibility and no skin irritant response of nanofibers in contact with a reconstituted three-dimensional human skin model, while scratch assay using human fibroblast cell line HDFa revealed the valuable potential of CS/PEO nanofibers to promote cell migration at an early stage of injury.

## 1. Introduction

Nanotechnology is a dynamically growing discipline with a large potential in the medical and pharmaceutical fields. Among a wide range of nanomaterials, including nanotubes, nanoparticles, and nanoflakes [1,2], nanofibers are a type of fiber-shaped structure characterized by large surface area and high interconnected porosity, which favor their exploitation as tissue scaffolds [3] or wound dressing materials [4]. The abovementioned properties can make nanofibrous materials structurally similar to the extracellular matrix in native tissues [5]. Nanofibers may be produced from natural and synthetic polymer, or from their combination. In particular, nanomaterials comprised of swellable biopolymers with profound ability to stimulate tissue proliferation have been of great interest in the field of wound treatment in recent years.

Electrospinning has been the most widely explored method of polymer-based nanofibers preparation thus far [6]. The process utilizes an electric field to control the formation of fibers. To simplify the fiber formation setup and eliminate potentially dangerous high voltage, solution blow spinning (SBS) has been recognized in recent years. SBS is a simple and efficient alternative technique for polymeric nanofiber fabrication [7,8].

Basically, in SBS, a polymer solution is flown through the inner nozzle and stretched into fibers by a pressurized airflow, enabling their deposition on a collector in the direction of gas flow. It is a robust process with a relatively high production rate and low energy consumption which, in contrast to the electrospinning method, does not need the use of harsh organic solvents or high voltage [9].

Among biopolymers used in spinning technologies, chitosan (CS) is a biocompatible polysaccharide composed of randomly ordered glucosamine and N-acetylglucosamine units linked with β (1 → 4) glycosidic bonds, and has attracted a particular attention [10]. CS is commercially produced in a deacetylation process of chitin attained mostly from exoskeleton of crustaceans, insects, mollusks, and cell walls of several fungi species [10,11]. CS is regarded as functional biopolymer with unique polycationic behavior, which enables interaction with negatively charged molecules and is responsible for its biological properties, including bioadhesiveness and antimicrobial activity [10,11,12]. Besides its biocompatibility, CS is known for its superior hemostatic activity, which mode of action is related to platelet activation and thrombin generation [13]. All these attractive features make CS interesting material for a number of biomedical applications including wound dressings [14], drug carriers [15,16], scaffolds for tissue engineering [17,18], and gene delivery platform [19]. 

It should be stated that CS is a relatively inhomogeneous material (particularly due to its origin and manufacturing process) and a wide range of CS types differing, e.g., in purity level, deacetylation degree, or molecular mass, are thus commercially available. These internal properties influence not only the final product characteristic or its applicability, but also may determine the suitability of a particular type of CS in a technological process. For instance, CS with higher molecular weight (above 200–250 kDa) can be considered as a viscous agent for semi-solid preparations (including hydrogels or emulgels) [20], whereas CS with low molecular weight (below 200–250 kDa) appears more appropriate for techniques where low viscosity dispersions are required, e.g., spray-drying [21], or microfluidic technology [22].

Poly(ethylene oxide) (PEO) is a class of nonionic synthetic polymers also applied in spinning techniques solely or as additives to other polymers [23]. PEO is considered biocompatible material with high solubility in an aqueous environment, good lubricity, and viscoelastic behavior—properties valuable in pharmaceutical technology, including tableting or coating [23,24,25]. Several papers describe the application of CS in combination with PEO in the electrospinning process [14,26,27]. Notably, the presence of PEO modulates surface tension and viscosity of acidic CS dispersion, making it more spinnable and facilitating the formation of electrospun nanofibers [28].

To our best knowledge, there are only a limited number of studies focused on the use of CS in solution blow spinning [29,30] and no research work devoted to the application of CS and PEO blends in SBS. In the present studies, a one-step SBS technique was therefore applied to produce nanofibers consisted of PEO and high-medical-grade CS. Designed formulations were characterized in terms of their application as wound care nanomaterial by structural and mechanical properties, as well as biocompatibility. Particularly important was to evaluate in vitro wound exudate absorption capacity and bioadhesive characteristics in contact with excised human skin in comparison to commercial wound hydrofiber-like materials. A potential to promote fibroblast migration by in vitro scratch assay was investigated and an irritation test using a three-dimensional EpiSkin tissue model was performed. Considering the issues related to the excessive adherence of conventional hydrofiber-like dressings and plausible damage of a wound bed and surrounded tissue upon their removal, the influence of additional coating with hydrophobic poly(dimethylsiloxane)–applied to favor removal of nanofibers from the wound surface—on the mechanical and bioadhesive behavior of nanofibers was examined.

## 2. Results and Discussion

### 2.1. Physicochemical Characteristics

**Figure 1:**
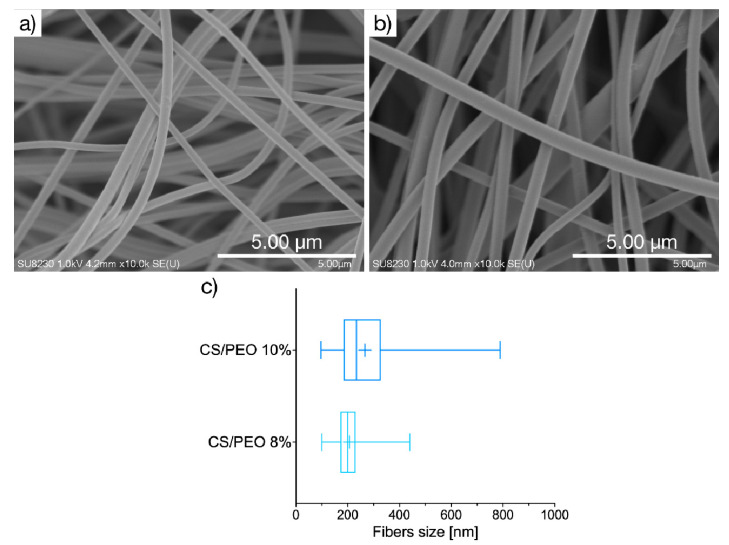
Representative SEM images of CS/PEO nanofibers prepared of: (**a**) 8% or (**b**) 10% (*w*/*w*) polymer blend solution (magnification ×10,000); (**c**) size distribution box-whiskers graph, where box represents, from the left: 25th percentile, median, and 75th percentile, with + sign for mean value, and whiskers represent minimum and maximum of fiber size distribution.

The current strategy in wound care treatment focuses on developing functional materials displaying some structural similarity to the extracellular matrix of native tissues and favoring the skin repair process. The unique properties of CS and PEO, including biodegradability and profound swelling capacity, led us to elaborate on their potential as components of solution blow-spun nanomaterial for skin healing and regeneration. In the present studies, SBS technique was for the first time applied to produce two nanofibrous materials consisted of PEO and CS in a mass ratio 4:1 from 8% (formulation CS/PEO 8%) or 10% (*w/w*) polymer solutions (formulation CS/PEO 10%). Morphological properties of CS/PEO nanofibers were investigated by using scanning electron microscopy. Basically, SEM imaging (Figure 1) demonstrated that fibers were randomly distributed to form a three-dimensional, highly porous structure. Both formulations CS/PEO 8% and CS/PEO 10% possessed a smooth surface and uniform structure of blended polymers with no cracks or beads along the fiber length. The chosen parameters allowed production of nanofibers with an average diameter of about 200 nm and 260 nm for CS/PEO 8% and CS/PEO 10%, respectively. Increasing the polymers concentration in solution for SBS process resulted in wider fiber diameter distribution (Figure 1c).

**Figure 2:**
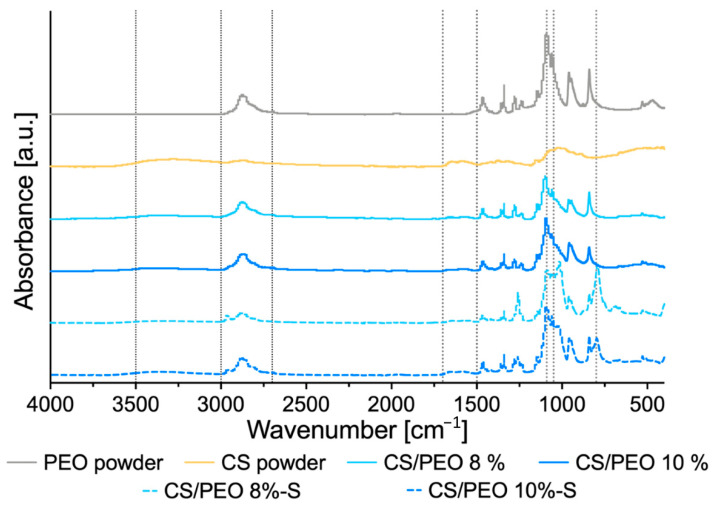
ATR-FTIR of pure poly(ethylene oxide) (PEO), chitosan (CS), uncoated nanofibers prepared of 8% (*w/w*), or 10% (*w/w*) CS/PEO solution and corresponding poly(dimethylsiloxane) modified nanofibers CS/PEO-S.

ATR-FTIR analysis was carried out to investigate PEO, CS, and poly(dimethylsiloxane) compatibility, and the plausible effect of SBS process on the interaction between PEO and CS. ATR-FTIR spectra of unmodified CS/PEO nanofibers and poly(dimethylsiloxane) modified nanofibers CS/PEO-S with respect to pure PEO and CS are shown in Figure 2.

The spectrum of pure PEO showed a characteristic absorption peak at 2885 cm^−1^ assigned to asymmetric stretching vibrations of -CH group and a prominent complex absorption intensity at around at 1050 and 1020 cm^−1^ attributed to C–O–C stretching mode [31]. In contrast, ATR-FTIR spectrum of pure CS displayed a broad peak at 3000–3500 cm^−1^ assigned to CS hydrogen bond stretching and a broad peak in the range from 1520 to 1700 cm^−1^, most probably corresponding to the N-H bending of the primary amine and the stretching of C = O bonds of amide in the presence of N-acetyl groups, respectively. Basically, spectral peaks of both tested uncoated nanofibrous materials were found comparable to those obtained from the pure PEO and CS sample and all characteristic peaks for each polymer were present in formulations CS/PEO 8% and CS/PEO 10%. No alterations in width and shape of peaks were observed, which pointed toward compatibility between polymers and indicated that the formation process of nanofibers did not influence the molecular nature of native polymers. S-coated formulations exhibited additional characteristic IR peaks resulting from the presence of poly(dimethylsiloxane) at 800 cm^−1^ (Si-C stretching), 1050 cm^−1^ (Si-O stretching), and 1260 cm^−1^ (deformation of CH_3_ in Si-CH_3_). In the region 1050 to 1090 cm^−1^ of spectra obtained for S-coated nanofibers, partially overlapping peak positions for PEO and poly(dimethylsiloxane) were noted.

**Figure 3:**
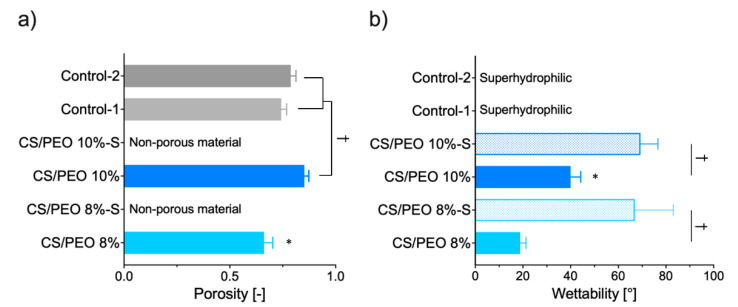
(**a**) Porosity and (**b**) wettability (water contact angle) of CS/PEO nanofibers as compared to poly(dimethylsiloxane) modified nanofibers CS/PEO-S and commercial hydrofiber-like wound dressings (Control-1; Control-2) (presented as the mean value ± standard deviation, *n* = 3). * represents significant differences with *p* ≤ 0.05 between uncoated formulations; † symbolizes differences with *p* ≤ 0.05 between uncoated nanofibers and (a) Controls or (b) S-coated formulations.

Uncoated formulations were characterized by relatively high porosity varied in the range from 75 to 85%, reflecting their good air permeability and breathability (Figure 3a). CS/PEO 10% exhibited a higher level of porosity when compared to the values obtained for commercial wound dressing materials prepared from carboxymethylcellulose sodium (Control-1) or calcium alginate (Control-2). Interestingly, in contrast to previously published studies, CS/PEO 8% material fabricated from solution with lower polymer concentration presented lower fiber packaging density [32]. After coating, nanofibers lost their porous structure (Figure 3a). SEM analysis of their surface morphology showed that fibers were fairly evenly covered with a poly(dimethylsiloxane) layer, which filled almost all free spaces in nanofibrous material (Figure S1).

In the healing process, it is important to remove excessive exudate fluid from the wound bed in order to facilitate and to speed up the reepithelization process [33]. Therefore, dressings with expanded surface area and high hydrophilicity are preferable. To investigate nanofiber hydrophilicity, water contact angle measurements were carried out. The effects of the polymer concentration applied in the SBS process and additional S-coating on the wettability of CS/PEO nanofibers were studied and compared to two commercial wound hydrofiber-like materials (Figure 3b). Uncoated nanofibers displayed a highly hydrophilic nature and absorbed water within the first second of the test. Thus, the water contact angle measurement was only possible for the first few seconds of the test. The higher concentration of polymer blend in solution for SBS resulted in a decrease in the hydrophilicity of formulation CS/PEO 10%. In turn, the applied Control-1 and -2 were wetted immediately, making it impossible to read the exact wettability value (and thus they were named as superhydrophilic materials). The additional coating slowed down but did not prevent complete surface wetting. The presence of a poly(dimethylsiloxane) layer resulted in almost two-fold and three-fold drops in the wettability values for formulation CS/PEO 10%-S and CS/PEO 8%-S, respectively. Notably, the obtained values of contact angle were below 90°, indicating that S-coated materials maintained their affinity for water and hydrophilic behavior.

### 2.2. Mechanical Properties and Absorption Capacity

**Figure 4:**
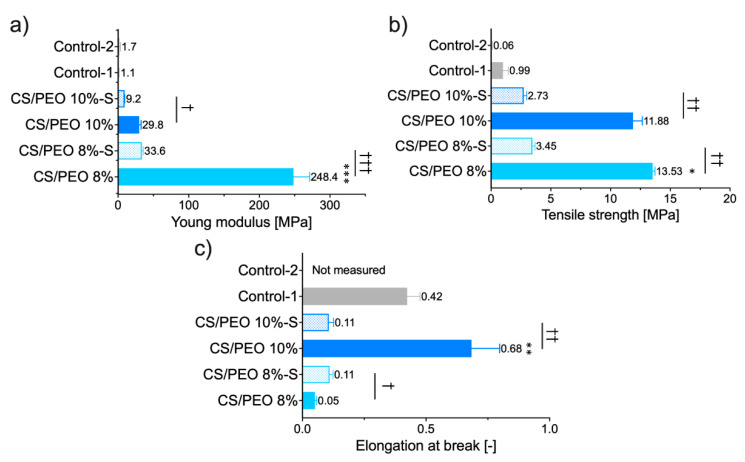
Mechanical properties of CS/PEO nanofibers before and after S-coating as compared to commercial wound dressings (Control-1; Control-2): (**a**) Young modulus, (**b**) tensile strength, and (**c**) elongation at break (mean ± S.D.; *n* = 5). Since the Control-2 sample was delicate in structure, the constant value of deformation (2 mm) was applied to estimate its Young modulus and tensile strength. * represents significant differences with *p* ≤ 0.05, while ** and *** represents significant differences with *p* ≤ 0.01 and *p* ≤ 0.001 between uncoated formulations; †, †† and ††† symbolize significant differences with *p* ≤ 0.05, *p* ≤ 0.01 and *p* ≤ 0.001 between uncoated and S-coated nanofibers, respectively.

Nanofibrous materials were next tested for mechanical properties, expressed as Young’s modulus (reflecting material stiffness), tensile strength (referring to maximum stress that the material can handle), and elongation at break, which displays its plausible ability to reversible deformation. The data from tensile measurements are displayed in Figure 4.

Profound differences among tested materials were noted and several features may be distinguished. The SBS process with polymer solution at concentration 8% (*w/w*) led to a significant increase in Young’s modulus of CS/PEO 8% material with simultaneous lower values of deformation compared to CS/PEO 10% (Figure 4). These properties point to the high stiffness of CS/PEO 8% formulation. Nanofiber materials′ structural and mechanical properties are mainly affected by membrane morphology, fiber packaging density, or fiber arrangement [34]. In our study, the greater stiffness of CS/PEO 8% material (when compared to CS/PEO 10%) was a consequence of two overlapping factors: its lower porosity (Figure 3) and reduced fiber sizes (Figure 1), which led to stronger interactions between fibers. Similarly, Conte et al. observed that the Young′s modulus of electrospun mats was reduced with an increased fiber density package [35], whereas Wong et al. found that poly(caprolactone) nanofibers with lower dimensions displayed an improvement in mechanical properties [36]. As in previously published papers [37], the randomly aligned fibers in CS/PEO samples appeared not to be the primary factor responsible for differences in stiffness between uncoated nanofibers as based on the SEM analysis, and both were found to be randomly aligned (Figure 1 and Figure S1). The low variations between replicates in tests with CS/PEO nanofibers samples (cut in different directions before measurements) pointed to their isotropic behavior and supported data from SEM analysis.

Formulation CS/PEO 10% exhibited the greatest values of elongation. Concerning wound dressing material, this feature enables the material to stretch and adjust to the desired shape more easily. Adjustment to the desired shape, in turn, may improve shape-conformation to the wound bed or impaired skin surface, making the material more comfortable to wear [38]. Still, it should be emphasized that more factors (including thickness, density, and tensile strength of the material) should be adequately tuned to guarantee comfort at the application site.

Both tested nanofibrous formulations were found more mechanically resistant and displayed profoundly higher values of tensile strength parameter in comparison to commercial wound dressings (Figure 4b). The observed ability to act as a physical barrier appears beneficial in terms of potential role in protecting from mechanical stress or environmental contamination. 

The effect of additional coating with poly(dimethylsiloxane), applied on the dressing material to favor its removal from the skin surface, on the mechanical properties of nanofibers was also examined. Interestingly, CS/PEO 8%-S displayed a slight increase in elongation at break value while a significant drop of this parameter was noticed in the case of the CS/PEO 10%-S sample. After S-coating, nanofibers showed markedly lower values of Young’s modulus and tensile strength (both by approximately one order of magnitude). Still, the attained parameters were found statistically higher than those obtained for commercial products (*p* < 0.05). Overall, it is clear that poly(dimethylsiloxane) significantly affected the mechanical behavior of designed SBS nanofibers. Among uncoated, CS/PEO 10% material shows better mechanical properties. Still, after S-coating, CS/PEO 8% with sufficient tensile strength and elongation ability appears more favorable and better fits to wound management. Based on these preliminary results, we cannot clearly select one formulation with the best mechanical behavior but still find them promising compared with tested commercial fiber-like dressings.

**Figure 5:**
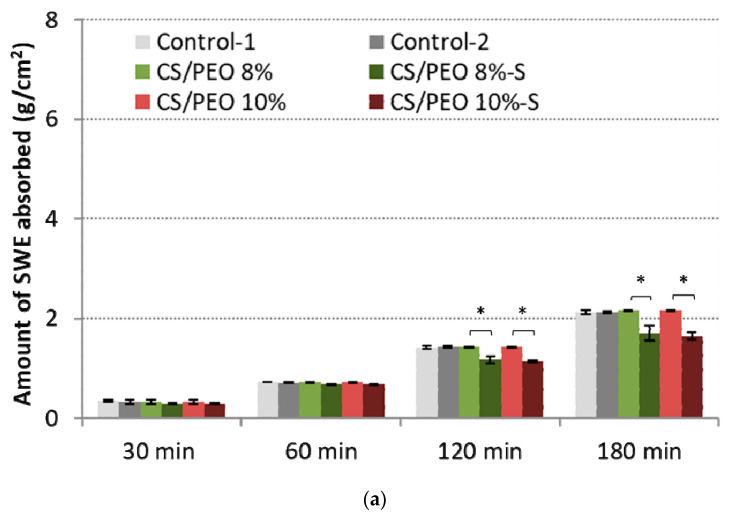

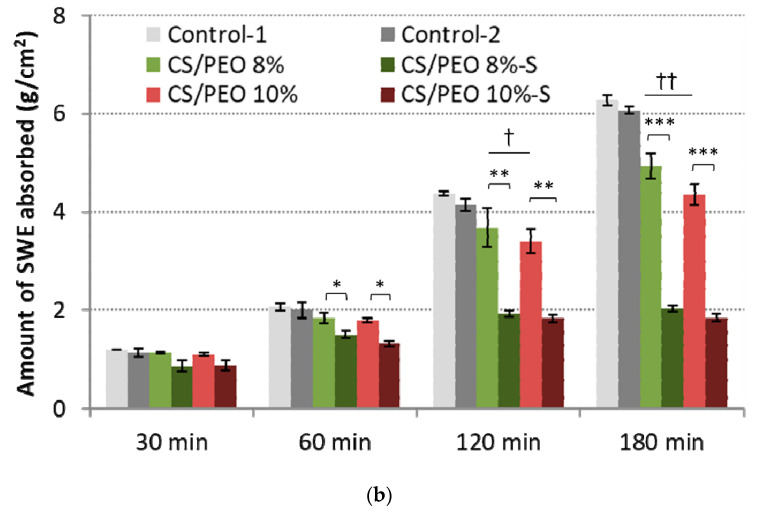
Total simulated wound exudate fluid absorption response of CS/PEO nanofibers and commercial wound dressings (Control-1; Control-2) at: (**a**) low (0.6 mL/h), and (**b**) high fluid flow rate (2.0 mL/h) (mean ± S.D.; *n* = 3); * represents significant differences with *p* ≤ 0.05, ** represents significant differences with *p* ≤ 0.01, while *** represents significant differences with *p* ≤ 0.001 between uncoated and S-coated nanofibers; † and †† symbolize significant differences with *p* ≤ 0.05 and *p* ≤ 0.01 between uncoated nanofibers and Controls, respectively.

The selection of optimal wound dressings is crucial in facilitating the healing process. Several classes of a wound can be distinguished. Depending on the amount of produced exudate, the classification includes low (shallow wounds, burns, or necrotic wounds), moderately (ulcers), to heavily exuding wounds (such as pressure injuries or skin maceration) [39]. To better understand the potential applicability of designed nanofibers, the absorption capacity was measured in vitro using SWE fluid (pH 7.8) in two variants simulating mildly or highly exuding wounds [40]. The results are presented in Figure 5.

All examined formulations maintained the intact structure thorough the test and no signs of disintegration or fiber loss were noticed. At the initial stage of the test, SWE was absorbed almost immediately by nanofibrous materials, which gradually transformed into a gel-like matrix. Despite the presence of a poly(dimethylsiloxane) layer, the efficient fluid uptake was noticed. No significant differences in absorption capacity with low SWE flow rate between uncoated and S-coated nanofibers were noted within the first 60 min of the test (Figure 5a). However, in the later stage of the test, the presence of the poly(dimethylsiloxane) layer was responsible for a reduced penetration of fluid into the CS/PEO network, and about 70% of total SWE fluid was uptaken by S-coated nanofibers. Uncoated nanofibers and controls absorbed more than 90% of SWE.

In the high-SWE flow rate measurements, profound differences between coated and uncoated materials were observed (Figure 5b). Formulations CS/PEO 8% and CS/PEO 10% had comparable SWE fluid uptake to commercial wound dressings and absorbed almost 90% of total fluid dropped on their surface within the first 60 min of the test, displaying satisfactory water uptake. Formulation CS/PEO 8% showed slightly higher absorbency when compared to CS/PEO 10% nanofibers. This observation is consistent with the results from wettability measurements (Figure 3b). In contrast, the poly(dimethylsiloxane) layer prevented fluid uptake, and S-coated nanofibers absorbed about 70% of SWE fluid at the same time. It should be noted that none of the tested S-coated CS/PEO nanofibers were able to efficiently absorb SWE fluid with an increased flow rate of SWE fluid in the later stage of the test (Figure 5b). After 2 h and 3 h, about 30% and 40% of SWE fluid dropped on the surface of the S-coated nanofibers was collected on the weight, suggesting the obtained materials could be instead applied on wounds with low exudates, including minor burns. In turn, uncoated CS/PEO nanofibers had the potential for application in moderate exudate wounds.

### 2.3. Bioadhesive Behavior

In developing wound dressing materials, it is vital to balance the level of bioadhesion, which should be adequate to adhere to the damaged skin and maintain the material in situ for a proper period. Still, simultaneously the material should not cause any damage to the surrounding tissue upon its removal [41,42]. CS, with intrinsic hemostatic effect, has been widely explored as functional biopolymer in the technology of wound dressings [43]. It is known for its relatively strong ability to interact with skin and mucosal tissue and can easily adhere to the impaired skin. This in turn may lead to undesirable effects in terms of its clinical application, e.g., upon its removal from the tissue [44]. To elaborate on the bioadhesive behavior of solution blow-spun CS/PEO nanofibers, ex vivo studies measuring the strength and work required to separate CS/PEO nanofiber materials from excised human skin material were carried out

**Figure 6:**
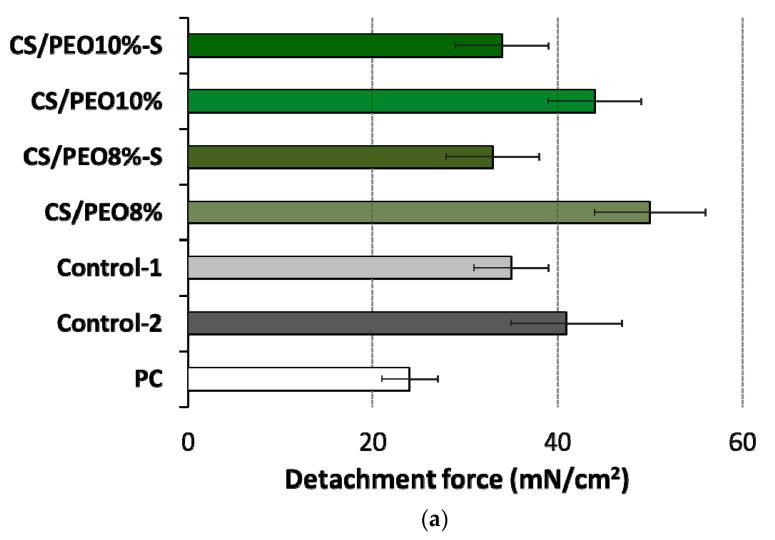

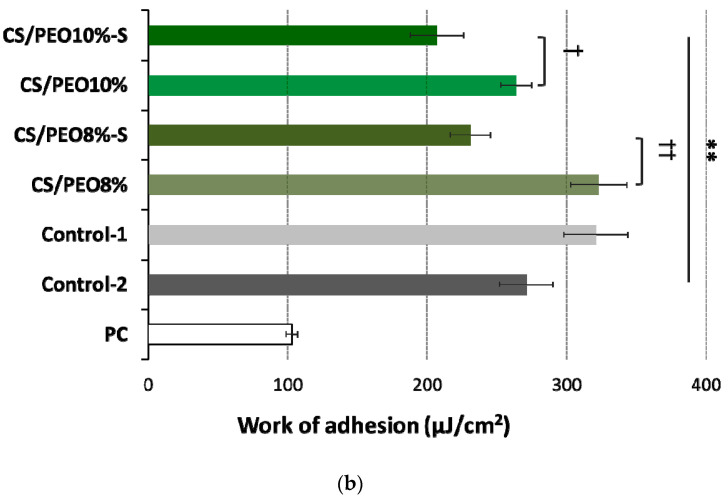
(**a**) Detachment force and (**b**) work of adhesion of CS/PEO nanofibers (prepared from 8% or 10% polymer blend solution) before and after S-coating as compared to cellulose paper (PC) and commercial wound dressings from carboxymethylcellulose sodium (Control-1) or calcium alginate (Control-2) (mean ± S.D.; *n* = 5); ** represents significant differences with *p* ≤ 0.01 in comparison to PC; † and †† symbolize significant differences between uncoated and S-coated nanofibers with *p* ≤ 0.05 and *p* ≤ 0.01, respectively.

In these measurements, a detachment force parameter referred to a mechanical stress, e.g., arisen from the body movements disturbing the contact between material and skin (Figure 6a). In turn, a work of bioadhesion described the overall ability to retain in position upon gradual material removal from the application site (Figure 6b). Nanofibers were found to interact with human skin compared to wetted cellulose film (discriminating the effect of cohesive forces on overall adherence) and displayed slightly higher bioadhesive strength values than those obtained for commercially available dressings (Control-1 and -2). Upon contact with tissue, the surface of wetted nanofibers transformed into a hydrogel matrix that favored the skin’s interaction.

No alterations in the level of bioadhesion force were noticed between CS/PEO 8% and CS/PEO 10% (Figure 6a), which suggests a relatively comparable resistance of these formulations to body movements. Formulation CS/PEO 10% (with an increase in total content of polymers in solution for SBS) showed lower values of work of bioadhesion of about 18% as compared to CS/PEO 8% formulation (Figure 6b). This observation suggests that CS/PEO 10% can be removed from the wound surface more easily. The effect of additional coating with poly(dimethylsiloxane) on nanofiber bioadhesiveness was also visible. In the case of formulation CS/PEO 8%, S-coating was responsible for a drop in force and work of bioadhesion of about 35% and 40%, respectively. In turn, nanofibers CS/PEO 10% were found less sensitive to bioadhesion changes upon S-coating and both examined parameters were about 20% lower in comparison to uncoated material.

### 2.4. In Vitro Cytotoxicity Studies and Irritant Test with EpiDerm^TM^

**Figure 7:**
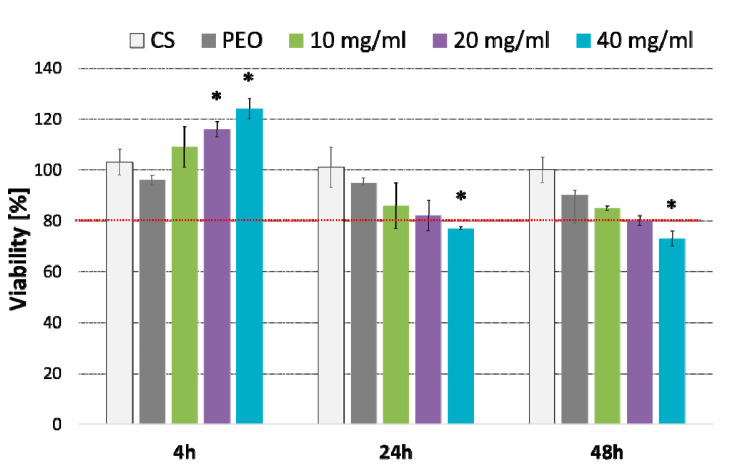
Column charts displaying viability of fibroblasts cells (as compared to untreated cells) incubated with different concentrations (expressed in mg/mL) of CS/PEO 10% nanofibers for 4, 24 or 48 h measured by MTT assay (mean ± SD; *n* = 3); cells treated with pure CS and pure PEO at concentrations corresponding to those in extract sample CS/PEO 10% at 40 mg/mL were used as controls. * represents significant differences with *p* ≤ 0.05 in comparison to untreated cells.

To assess the safety profile of designed nanofibers, MTT and JC-1 tests in human fibroblast cells (HDFa) were carried out. Based on the obtained data, nanofiber CS/PEO 10%, with adequate mechanical properties, moderate bioadhesiveness, and satisfactory absorption capacity, was selected for the in vitro cytotoxicity studies. The effect of nanofiber extract (prepared according to [45]) on the fibroblasts viability tested by MTT assay is presented in Figure 7.

Basically, nanofiber extract in concentration 10 and 20 mg/ml exhibited no effect on the cell viability over 48 h incubation. When compared to untreated cells, the highest concentration 40 mg/ml caused a slight decrease in cell viability (23% and 26% after 24 and 48 h exposure, respectively) (*p* < 0.05). Interestingly, a substantial increase in metabolically active cells (in respect to untreated cells) was observed after the first 4 h of incubation with nanofiber extract (*p* < 0.05). This increase in metabolic activity of the HDFa cell line was particularly visible in cells incubated with its highest concentration and suggest the plausible impact of nanofibrous material on fibroblast growth.

**Figure 8:**
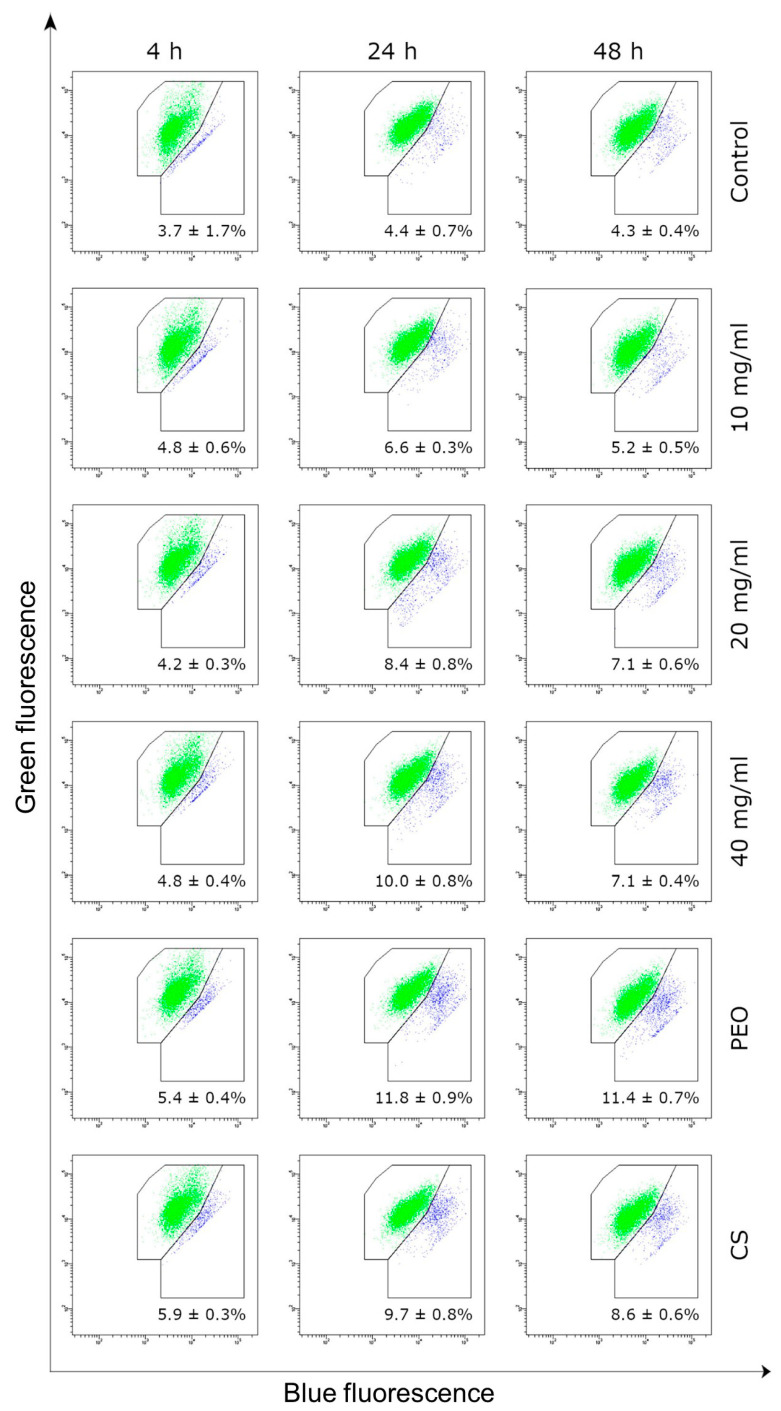
Representative dot-plots displaying the mitochondrial membrane potential of HDFa fibroblast cells incubated for 4, 24 or 48 h with CS/PEO 10% extract in concentration range 10–40 mg/mL measured by JC-1 fluorescence (green-cells with normal MMP, blue-with decreased MMP); data embedded in each dot-plot (as mean for three independent measurements) represents the percent of cells with decreased MMP; untreated cells (K0), cells treated with pure CS and pure PEO at concentrations corresponding to those in sample CS/PEO 10% at 40 mg/mL were used as controls.

Cytotoxicity was additionally tested by the fluorescent mitochondrial assay that detects the loss of mitochondrial membrane potential (MMP). The influence of CS/PEO 10% nanofibers on the MMP of fibroblasts after 4, 24, and 48 h incubation is presented in Figure 8.

The JC-1 assay showed no substantial effect of nanofibrous material on the MMP of fibroblasts cells upon 4 h exposure (as compared to untreated cells). Nanofiber extract stimulated the loss in mitochondrial membrane integrity in a concentration-dependent manner after 24 h and 48 h incubation, though the observed decrease in MMP did not exceed 10% in any of tested samples. Interestingly, the HDFa cell line was more sensitive to the presence of pure PEO sample and the percentage of cells with the loss of MMP was about 12% after 24 h incubation. It should be stated that a steady return to primary values of MMP was observed in cell samples treated with all concentrations of nanofiber extract after 48 h. This may be related to the subsequent proliferation of cells in time, which in turn compensated the total level of cells with lost MMP. Obtained data pointed out that CS/PEO nanofibers obtained through the SBS process exerted no significant impact on the fibroblast cell viability over 48 h incubation. 

CS/PEO 10% nanofibrous material was additionally evaluated for its irritation potential in contact with the three-dimensional highly differentiated tissue EpiDerm [46,47]. The material was added (in an amount of 25 mg) to MTT medium and incubated at 37 ± 1 °C, 5% CO2, for 60 min. According to the EU and GHS classification (R38/Category 2 or no label), an irritant potential is predicted for samples when the mean relative tissue viability of three individual tissues exposed to the tested material is reduced below 50% of the mean viability of the negative control [48]. The viability was decreased to 2.5% by SDS but was not altered in the presence of nanofibers (Table 1). CS/PEO 10% nanofibrous material displayed comparable results to those obtained for negative control (PBS treated tissue), confirming its non-irritancy and compatibility with the skin model.

The performed test met the acceptance criterion as the mean viability of positive control tissues expressed as % of the negative control tissues was ≤20% and standard deviation calculated from individual % tissue viabilities of the three identically treated replicates was below 18%.

### 2.5. In Vitro Scratch Assay

**Figure 9:**
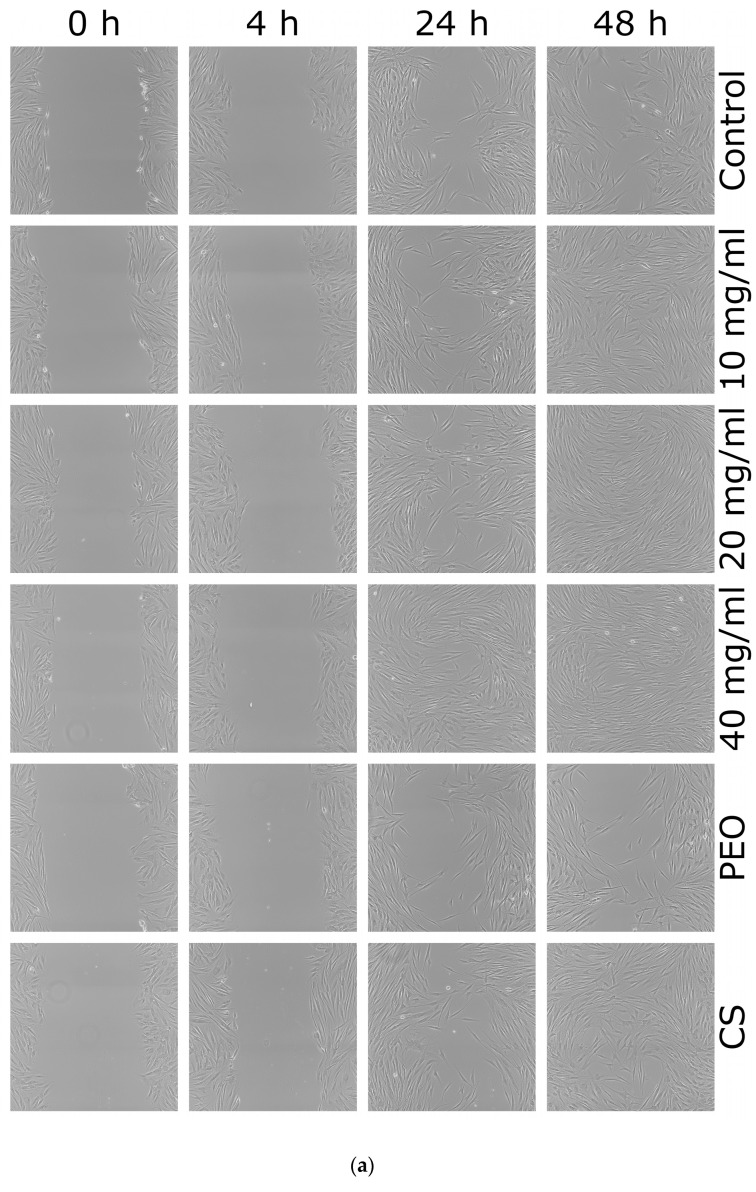

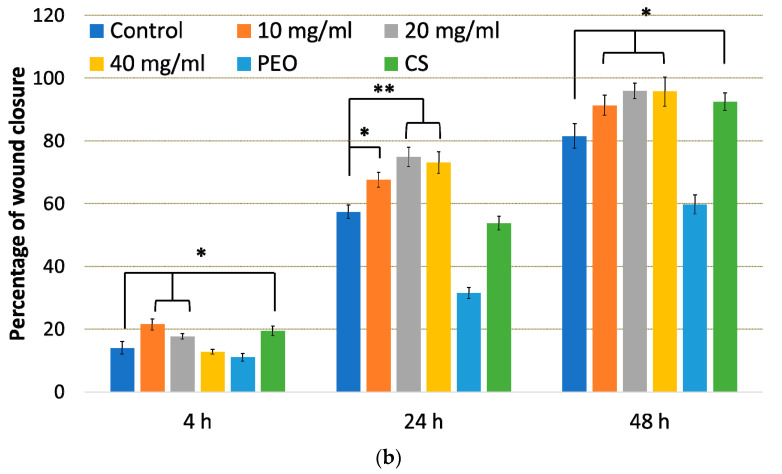
(**a**) Representative scratch assay images and (**b**) percentage of wound closure over 48 h period representing cellular migration and proliferation of control cells, cells in the presence of: nanofiber CS/PEO 10% extract at concentrations 10, 20 and 40 mg/mL, pure PEO, or pure CS as controls. * represents significant differences with *p* ≤ 0.05, while ** with *p* ≤ 0.01 in comparison to untreated cells.

Wound healing is a complex process comprising partially overlapping stages: inflammation, proliferation, and remodeling. In all phases, both immune and epithelial cells (fibroblasts and keratinocytes) are important cells that play a vital role as structural support in wound repair and trigger the immune cells. CS is regarded as a promising compound in wound treatment. It modulates the wound-healing process, e.g., by accelerating the formation and differentiation of stromal cells (fibroblasts and keratinocytes) and stimulating growth factor release [49,50]. In vitro scratch assay was applied to elaborate the apparent effect of the SBS process on the biological activity of CS in a CS/PEO nanofiber mat. Data from an in vitro scratch assay enabling observation of cell migration [51] after contact with nanofiber extract are presented in Figure 9.

From these images, it is clear that either pure CS and all concentrations of nanofiber extract promoted healing properties and complete scratch closure was observed over the time period of 48 h in contrast to the control group in which the scratch mark was still visible after 48 h. Cellular proliferation and migration processes were faster in the presence of nanofiber extract 10 mg/ml and pure CS where positive healing properties were noticed at initial time points. In contrast, the presence of pure PEO slowed down the “healing” process and prevented complete scratch closure upon 48 h. After 4 h incubation, the percentage of wound closure in HDFa cells treated with nanofiber extract at 10 mg/ml was higher as compared to control cells (Figure 9b). In turn, cell incubation with extract at 20 and 40 mg/ml resulted in almost 80% scratch closure after 24 h. This observation indicates that CS accelerates wound healing at an early stage of the injury. Similarly, Huang et al. found that CS regulates the inflammation–proliferation transition at the cellular level by reducing the inflammatory phase (e.g., stimulating macrophages and stromal cells) and speeding up the proliferation stage [50]. Studies performed by Howling et al. indicated that enhanced fibroblast proliferation (involved in regulating the level of inflammation) in the presence of CS resulted from polyelectrolyte complex formation with macromolecules (including growth factors) present at a wound site [49]. 

The obtained data are consistent with previously published papers devoted to CS-based wound-healing materials and pointed out that CS preserved its biological activity towards fibroblasts during the SBS process. Correspondingly, Bagheri et al. showed that electrospun CS/PEO nanofibrous mats increased fibroblast migration after 24 h exposure [52]. Several research papers also presented the effect of CS-based materials on keratinocytes involved in the re-epithelization process. For instance, in studies on microporous tissue scaffold, Salerno et al. showed that CS-based membrane facilitates proliferation, stratification, and migration of keratinocyte cells [53]. In turn, electrospun CS/lignin/PEO fibrils were found to modulate the expression of pro-inflammatory cytokines (interleukins -1 and -8, tumor necrosis factor-α) and matrix metalloproteinases in keratinocyte cells, suggesting an anti-inflammatory and immunomodulatory role, promising on the restoration of damaged tissues [54]. 

In this study, we presented that solution blow-spun CS/PEO nanofibers per se are capable of initiating the healing and regeneration process at very early stage of injury.

## 3. Materials and Methods

### 3.1. Materials

Highly purified medical grade chitosan (Chitoscience^®^) was purchased from Heppe Medical Chitosan GmbH (Halle, Germany). The average molecular weight (232 kDa) was assessed by Agilent 1260 Infinity GPC/SEC at 35 °C with a refractive index detector (Agilent Technologies, Santa Clara, CA, USA) The exact value of deacetylation degree (79.5%) was determined by titration method according to [55]. Polyethylene oxide (PEO, M_w_ = 900,000 g/mol) was purchased from Sigma Aldrich (Darmstadt, Germany) and poly(dimethylsiloxane) was from Fagron (Kraków, Poland). Commercially available hydrofiber-like wound dressings comprised of calcium alginate (Sorbalgon, Hartmann, batch 2870724) and from carboxymethylcellulose sodium (Aquacel, ConvaTec, batch 5L10879) were used as controls for mechanical and bioadhesive tests. MTT [(3-4,5-dimethyl thiazole 2-yl) 2,5-diphenyltetrazoliumbromide] was purchased from Sigma and JC-1 MitoScreen kit was obtained from BD Biosciences (San Diego, CA, USA). EpiDerm tissue (lot number 28690) was from MatTek In Vitro Life Science Laboratories (Bratislava, Slovak Republic). Simulant wound exudate fluid (SWE, pH 7.8, viscosity 400 mPas at ambient temperature) for in vitro absorption capacity measurements and bioadhesive studies was prepared according to [56] with the following composition (g per 100 g of water): sodium chloride, 4.15; calcium chloride, 0.18; and hydroxyethylcellulose, 4.0. The additional gelling agent was selected upon preliminary studies to simulate the viscous behavior of exudate. All other chemicals were obtained from Chempur (Piekary Śląskie, Poland).

### 3.2. Preparation of Polymer Blend Solutions for Blow Spinning

Suitable concentration and ratio of PEO and CS for the fiber formation process were selected upon preliminary studies. Solutions with blend of polymers containing 1.6 and 2% (*w*/*w*) CS and 6.4 and 8% (*w*/*w*) polyethylene oxide (PEO) were dissolved in 0.5% (*v*/*v*) acetic acid aqueous solution and left for continuous stirring for 24 h in room temperature. Overall concentration of polymers in the solution was 8 and 10% (*w*/*w*). Concentration below 8% had too low viscosity whereas concentration above 10% was found to clog nozzle during SBS process. According to [57], the lowest concentration of acetic acid necessary to complete CS dispersion was used.

### 3.3. Solution Blow Spinning Technique

Nanofibers were produced in SBS process, described in detail previously by our group [58]. During preliminary assessment the air pressure, the polymer solution feed rate, and working distance between collector and nozzle were suited to assure stable spinning process. Briefly, freshly prepared polymer blend solution was supplied through the inner nozzle (diameter 0.8 mm) of the concentric nozzles system in the SBS apparatus in closed cabinet under ambient temperature and at 35–40% of relative humidity. The flow rate of polymer solution was adjusted according to concentration of polymer blend solution and varied in the range from 0.5 (for 8% (*w*/*w*)) to 1.0 mL/h (10% (*w*/*w*)). At the same time, the airstream was supplied through the outer nozzle of the SBS nozzles system with a constant pressure of 0.08 MPa. Fibers were produced by shear-drag elongation of the polymers blend solution by the stream of air at the distance of 50 cm between the nozzle system and the surface of the collector. As a collector, the rotating cylinder with reciprocating motion was used (cylinder diameter: 10 mm, length: 100 mm, rotational speed: 3000 rpm). The process continued until the formation of a nanofibrous mat with a thickness of approximately 100–200 μm and was carried out three times for each nanofibrous material’s composition.

For mechanical and bioadhesive measurements, nanofibrous materials were additionally coated with liquid poly(dimethylsiloxane) (S-coating) which was sprayed uniformly through a nozzle (diameter 0.8 mm) on their surface in a mass ratio 1:1. The distance between the nozzle and the nanofibers was 10 cm. 

### 3.4. Scanning Electron Microscopy

The morphology and fiber size were investigated using scanning electron microscopy (SEM, SU8320, Hitachi, Tokyo, Japan). Samples were prepared by cutting a mat into a 5 mm × 5 mm square sample and putting it on the surface of the SEM stub using carbon/aluminum conducting tape. Before imaging, samples were coated (Q150T, Quorum, Laughton, UK) with about 10 nm layer of Au:Pd (80:20 atomic ratio). For each sample at least 100 fibers were measured in three different areas of observation.

### 3.5. Porosity

The porosity of the nanofibrous materials was determined using a gravimetric method based on the following relation:

P(%) = (1 − d_s_/d_p_) × 100% (1)

where d_s_—sample density, d_p_—polymer density. Square samples with dimensions about 10 mm × 10 mm were weighed, and sample density was calculated: d_s_ = m/(δ∙A), where m—sample mass (g), δ—sample thickness (cm), A–sample area (cm^2^) [59]. The sample thickness was measured using SEM. Porosity measurement was performed three times, and the values were expressed as mean ± standard deviation. 

### 3.6. Wetting Properties

Hydrophilic properties of CS/PEO nanofibers were studied using the sessile drop method (DSA100 goniometer, Krüss GmbH, Hamburg, Germany). Droplets of 5 μL distilled water were dispensed onto the surface of the material (with area 1.0 cm^2^) at ambient conditions, and the water contact angle was measured over 1 s with 10 frames per second. The water contact angle for each material was measured three times, and values are presented as mean ± standard deviation.

### 3.7. Attenuated Total Reflection Fourier Transformed Infrared Spectroscopy (ATR-FTIR)

The presence of the specific functional groups of each compound in CS/PEO nanofibers was determined by ATR-FTIR Nicolet™ 6700 spectrometer (Thermo Fisher Scientific, Waltham, MA, USA). Spectra were detected three times in ATR mode and analyzed with the OMNIC 8.3 software, and representative spectrum for each sample is presented as a result.

### 3.8. Mechanical Characteristics

Rectangular samples of CS/PEO nanofibrous materials underwent a uniaxial stretching test according to previously described protocol [7]. For this purpose, samples of CS/PEO 8% and CS/PEO 10% formulations before and after coating with poly(dimethylsiloxane) were randomly cut along and across to exclude the influence of fiber orientation on tensile behavior. Additional controls, commercial hydrofiber-like wound dressings (cut only on the fiber length) comprised of carboxymethylcellulose sodium (Control-1) and calcium alginate (Control-2) were applied.

The test was conducted using Instron 3345 model (Norwood, MA, USA) with crosshead speed 10 mm/min at ambient conditions which measured the maximum load and strain at rupture. Each experiment was carried out five times.

SEM imaging and porosity measurements displayed that fibers occupy only a fraction of the volume of the material sample. One can determine this fraction from the 1-P relationship, which also applies to the cross-sectional area of the sample. Since only a fraction of fibers occupies the cross-sectional area of the sample, only this fraction of the material thickness transfers the applied load during tensile testing. This effect was accounted for in the data processing for tensile properties calculation. Due to poor mechanical properties of Control-2, fixed distance at which the probe was raised (about 2 mm) was applied to estimate its tensile strength and Young modulus. 

### 3.9. Simulate Wound Exudate Absorption Capacity

Absorption capability was measured using simulate wound exudate (SWE) fluid on a self-constructed thermostated inclined steal plate at 32 ± 1 °C according to [60,61]. Each sample was adhered to horizontally positioned plate which was next set at 45° inclination in order to simulate changing in body position. SWE fluid was then carefully dropped on the surface of the material at determined time intervals with a constant rate of 0.6 or 2 mL/h (according to [40] simulating low and high flow rate, respectively). The amount of SWE not absorbed by tested material was weighed thorough the study (Radwag XA 60/220, Radom, Poland). At the end point of the test, capacity of the dressing to uptake fluid was determined by weighting sample. Results were expressed as amount of fluid absorbed per unit of material surface area. Each experiment was carried in triplicate.

### 3.10. Bioadhesive Behavior in Contact with Excised Human Skin

The protocol of bioadhesion study was approved by the Local Bioethical Committee, Bialystok, Poland (permission number R-I-002/305/2019). Freshly excised human skin was obtained from the clinic of surgery and aesthetic medicine in Bialystok from women undergoing face lift. Tissue specimens were preserved in the isotonic saline solution, frozen at −20 °C directly after the surgery and kept no longer than 60 days. In prior experiments, skin was thawed at ambient conditions, cut into pieces, and microscopically checked for tissue integrity.

Bioadhesive properties of nanofibers in contact with excised human skin were carried out with texture analyzer TA.XT. Plus (Stable Microsystems, Godalming, UK) [62]. Uncoated or S-coated with poly(dimethylsiloxane) nanofibers were adhered to the platform A/MUC and wetted with SWE (pH 7.8) whereas the skin sample was fixed with cyanoacrylate glue to the upper tester probe. Afterward, the probe was lowered onto the surface of nanofibers (with S-coated side facing towards the skin sample) with a constant speed of 0.5 mm/s and a contact force of 0.2 N for 60 s. Next, the materials were separated at a constant rate 0.1 mm/s. The strength of bioadhesion was recorded directly from Texture Exponent 32 software and the work of bioadhesion expressed in µJ per tissue area was measured from the area under the force vs. distance curve. Cellulose film and commercially available hydrofiber-like dressings served as controls. Each sample was tested using skin specimens excised from three donors and the results were presented as mean from five independent measurements. 

### 3.11. Cell Culture and Treatment

Human dermal fibroblasts cell line HDFa from American Type Culture Collection (Manassas, VA, USA) were propagated according to the enclosed procedure protocol in Dulbecco’s modified Eagle’s medium (DMEM) supplemented with 10% fetal bovine serum, 50 U/mL penicillin and 50 μg/mL streptomycin at 37 °C in an atmosphere with 5% CO_2_. The cells were seeded into six-well plates (with density of 5 × 10^5^ cells per well) and cultured for 48 h in optimal growth conditions (37 °C, 5% CO_2_). Extraction of CS/PEO 10% nanofibrous material in serum-free medium at concentrations 10, 20, 40 mg/mL took place at 37 °C for 72 h according to ISO EN 10993-5:2009 [45]. After filtration through 0.2 cellulose filters, extracts were carefully added to cell culture. The concentration range was chosen to exclude the impact of viscosity parameter on cell morphology, mobility and functions [63].

### 3.12. Cytotoxicity Assays

#### 3.12.1. MTT Assay

According to Carmicheal et al. [64], the viability assay was quantitatively measured by converting tetrazolium salt (MTT), present in cell mitochondria, into a blue formazan salt after extraction from the cell medium. The extracts of CS/PEO 10% nanofibers were added to 24-well plates containing confluent HDFa cells and incubated for 4, 24 or 48 h at 37 °C in a 5% CO_2_ humidified atmosphere. Then the culture medium was discarded, cells were rinsed three times with Phosphate Buffered Saline (PBS) and incubated for 4 h in MTT solution at 37 °C. Afterwards, medium was removed from the wells and the cells were lyzed with DMSO and Sorensen’s buffer. The absorbance of purple formazan derivative in living cells was measured spectrophotometrically at 570 nm (Jasco V 750, Abl&E Jasco, Kraków, Poland). Cell viability cultured in the presence of nanofibers extracts was calculated as a percent of control cells not exposed to CS/PEO material. Additional control, pure PEO and pure CS were dispersed in medium at concentrations 32 mg/mL and 8 mg/mL, respectively-referring to those occurred in the highest tested concentration of nanofiber extract. Experiments were performed for at least three independent replicates.

#### 3.12.2. JC-1 Assay

Disruption of the mitochondrial membrane potential (MMP) was analyzed using the lipophilic cationic fluorochrome (JC-1 MitoScreen kit) as previously described [15]. Subsequently, extracts of CS/PEO 10% nanofibers prepared at concentrations 10, 20 or 40 mg/mL were added to six-well plates and the cells were incubated for 4, 24 and 48 h. Next the unfixed cells were washed with PBS, suspended in PBS containing 10 mg/mL of JC-1, incubated for 15 min at room temperature (with simultaneous protection from light), then washed and resuspended in PBS. Afterwards, analysis was carried out immediately by flow cytometry BD FACSCanto II software (BD Biosciences Systems, San Diego, CA, USA). The percentage of cells with disturbed MMP was calculated using FACSDiva software (BD Biosciences Systems, San Diego, CA, USA).

#### 3.12.3. EpiDerm™ Skin Irritation Test

The test consists of a topical exposure of nanofibrous material to a reconstructed human epidermis model EpiDerm™ followed by a cell viability assay which was measured by dehydrogenase conversion of MTT. Test was performed according to the enclosed procedure described in Section 7.4. of EPI-200-SIT Protocol [65,66]. Briefly, after 60 min exposition to 25 mg of nanofibrous material CS/PEO 10% (37 ± 1 °C, 5% CO_2_, 95% relative humidity), tissues were thoroughly rinsed with PBS, blotted to remove the tested material, and transferred to fresh medium. After 42 h-incubation period, the MTT assay was carried out by transferring the tissues to 24-well plates containing MTT medium at a concentration of 1 mg/mL. After 3 h incubation, the blue formazan salt formed by cellular mitochondria was extracted with 2.0 mL/tissue of isopropanol and the optical density of the extracted formazan was determined spectrophotometrically at 570 nm. Sterile PBS was used as negative control whereas 5% (*w*/*w*) SDS solution was used as positive control and examined concurrently with nanofibers. Test was performed in triplicate.

### 3.13. In Vitro Scratch Assay

The scratch test was used to evaluate the migration ability of fibroblast HDFa cells in the presence of CS/PEO 10% nanofibers extract and pure CS or PEO controls. Cells were cultured in six-well plates until approximately 100% confluence. Thereafter, vertical scratches were produced using 20 μL pipette tips [51]. After washing the cells with PBS to remove the scraped cells, the fibroblasts were treated with nanofibers extracts in concentration range 10–40 mg/mL and incubated at 37 °C with 5% CO_2_. Images were taken after 4 h, 24 h and 48 h incubation using a phase contrast microscope (Nikon Eclipse Ti, Tokyo, Japan) at a 100× magnification. The wound closure expressed as a change in pixels intensity between cell monolayer and scratch area over time was assessed by ImageJ software (Version 1.53q, Bethesda, MD, USA) and calculated as a percentage of the initial scratch area at 0 h.

### 3.14. Statistical Analysis

The normality of fiber size distributions, porosity, water contact angle and results from mechanical and bioadhesive properties was tested using the Shapiro-Wilk test (*p* < 0.05). The difference among the mean fiber sizes, mean water contact angle values and bioadhesive behavior of analyzed materials were tested using the Kruskal-Wallis test with post hoc nonparametric Dunn’s test for multiple comparisons. The difference among the mean porosity and mean values of the mechanical properties of analyzed materials was tested in one-way ANOVA with post hoc Tukey’s test for multiple comparisons.

## 4. Conclusions

The SBS technique was successfully applied to fabricate porous nanomaterial consisting of PEO and high-medical-grade CS with uniform, beadless nano-sized fibers. The applied changes in concentrations of polymer blend used for SBS impacted fiber diameter, as well as stiffness, elasticity, and wettability of obtained nanofibers. Upon contact with simulated wound exudate, designed formulations formed a gel-like matrix, which appears necessary for a proper healing process. In vitro wound exudate absorption studies indicated their potential as dressings for wounds with low or moderate exudates. All tested materials displayed moderate adherence to an excised human skin surface, which decreased after coating with poly(dimethylsiloxane). The presence of a hydrophobic layer impacted mechanical properties of nanofibers, though the obtained values were comparable to those attained for commercial hydrofiber-like materials comprised of carboxymethylcellulose sodium or calcium alginate. CS/PEO 10% nanofibers exhibited cytocompatibility at concentration 10–20 mg/ml and fibroblast viability comparable to the control group at all tested time points. Solution blow-spun material did not induce irritancy in contact with EpiDerm™ tissue, which confirmed its compatibility with a skin model. Additionally, an in vitro scratch assay demonstrated its ability to promote fibroblast migration even at early time points. Overall, obtained results show that CS-enriched, solution blow-spun PEO nanofibers with a poly(dimethylsiloxane) outer layer may serve as valuable material per se for skin healing and regeneration. 

## Figures and Tables

**Table 1:** Data from EpiDerm Skin Irritation assay (mean ± S.D.; *n* = 3).

Sample	Viability ± S.D. (%)	Coefficient of Variance (%)	In Vivo Prediction
PBS (negative control)	100.0 ± 7.0	7.0	Non-irritant
5% SDS (positive control)	2.5 ± 0.3	10.5	Irritant
CS/PEO 10%	100.2 ± 2.2	2.2	Non-irritant

## Supplementary Materials

The following supporting information can be downloaded at https://www.mdpi.com/article/10.3390/ijms23095135/s1.
