# Supplementary material for: Chitosan-Enriched Solution Blow Spun Poly(Ethylene Oxide) Nanofibers with Poly(Dimethylsiloxane) Hydrophobic Outer Layer for Skin Healing and Regeneration
Source: Int J Mol Sci. 2022 May 5;23(9):5135. doi: 10.3390/ijms23095135 (PMC9105710; doi:10.3390/ijms23095135)
Supplement: Supplementary file 1 [file ijms-23-05135-s001.zip › ijms-1664982-supplementary.pdf]

SUPPLEMENTARY MATERIAL

Article

Chitosan-enriched solution blow spun poly(ethylene oxide) nanofibers with poly(dimethylsiloxane) hydrophobic outer layer for skin healing and regeneration

Emilia Szymańska [1,*], Michał Wojasiński [2], Robert Czarnomysy [3], Renata Dębowska [4], Iwona Łopianiak [2,5,7], Kamil Adasiewicz [6], Tomasz Ciach [2,7], and Katarzyna Winnicka [1]

[1] Department of Pharmaceutical Technology, Medical University of Bialystok, Mickiewicza 2c, 15-222 Białystok, Poland; e-mail: emilia.szymanska@umb.edu.pl (E.S.), kwin@umb.edu.pl (K.W.)

[2] Faculty of Chemical and Process Engineering, Warsaw University of Technology, Waryńskiego 1, 00-645 Warsaw, Poland; e-mail: michal.wojasinski@pw.edu.pl (M.W.); tomasz.ciach@pw.edu.pl (T.C.);

[3] Department of Synthesis and Technology of Drugs, Medical University of Bialystok, Kilińskiego 1,15-089 Bialystok, Poland; E-mail: robert.czarnomysy@umb.edu.pl (R.C.)

[4] Dr Irena Eris, Centre for Science and Research, Armii Krajowej 12, 05-500 Piaseczno, Poland; e-mail: Renata.Debowska@DrIrenaEris.com (R.D.)

[5] Doctoral School no. 1, Warsaw University of Technology, Plac Politechniki 1, 00-661 Warsaw, Poland; e-mail: iwona.lopianiak.dokt@pw.edu.pl (I.Ł.)

[6] Student Scientific Group at Department of Pharmaceutical Technology, Medical University of Bialystok, Mickiewicza 2c, 15-222 Białystok, Poland; e-mail: adasiewicz.k@outlook.com (K.A.)

[7] CEZAMAT, Warsaw University of Technology, Poleczki 19, 02-822 Warsaw, Poland

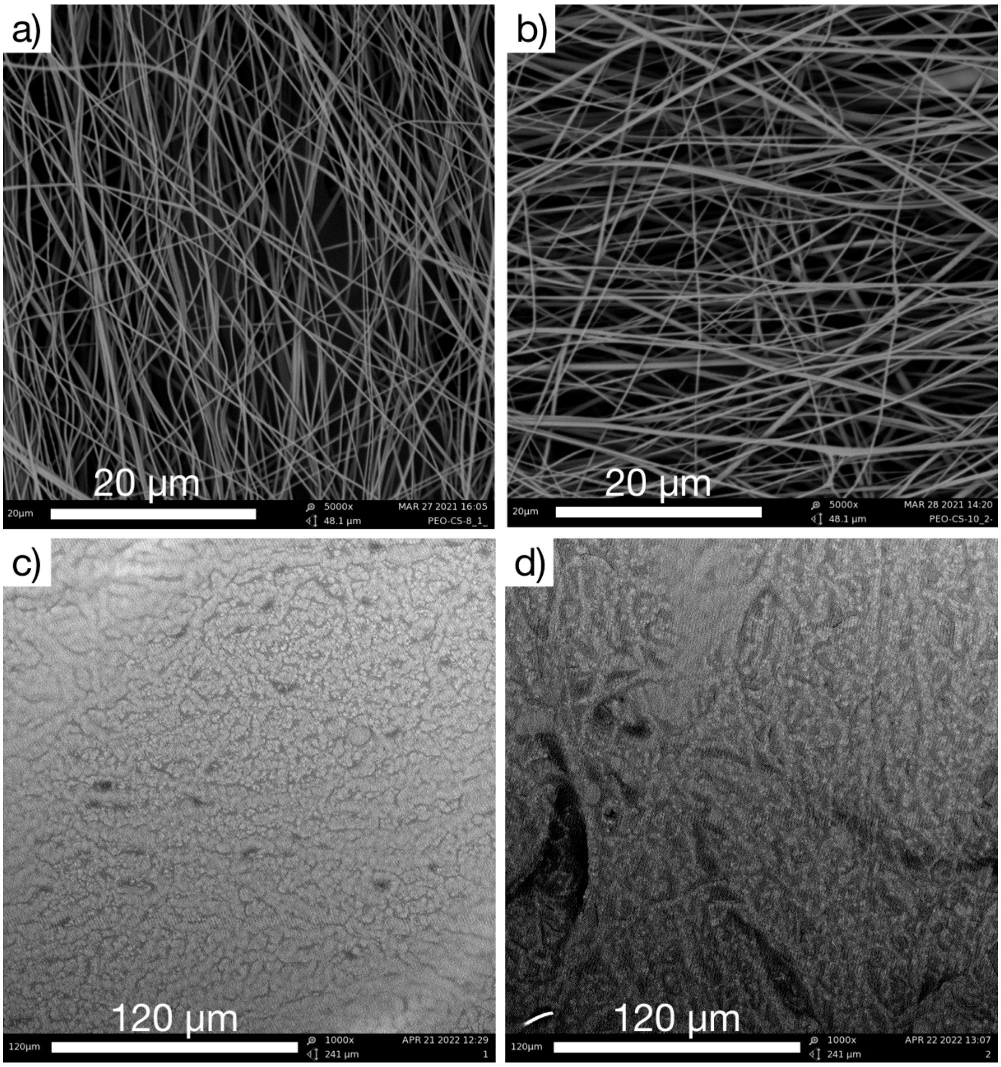

Figure S1. Representative SEM image of unmodified CS/PEO nanofibers prepared of: (**a**) 8% or (**b**) 10% (*w*/*w*) polymer blend solution (original magnification ×5000) and corresponding formulations upon coating with poly(dimethylsiloxane) (**c**,**d**) (magnification ×1000.
